# Association between the three-dimensional facial shape and its color in a boundary group of young to middle-aged Asian women

**DOI:** 10.1016/j.heliyon.2024.e32033

**Published:** 2024-05-28

**Authors:** Chihiro Tanikawa, Haruna Yamanami, Megumi Nagashima, Seiko Matsumoto

**Affiliations:** a Department of Orthodontics and Dentofacial Orthopedics, Osaka University Dental Hospital, Suita, Osaka, Japan; b MIRAI Technology Institute, Shiseido Co., Ltd., Yokohama, Japan

**Keywords:** Face, Humans, Women, Color, Aging

## Abstract

Visual cues strongly influence an individual's self-esteem and have fundamental sociopsychological functions. The color and shape of the face are important information for visual cues and are hypothesized to be correlated with each other. However, few studies have examined these relationships. Thus, this study determined the association between color and shape of the face. For this purpose, we evaluated Chinese women in their 30s and 40s (n = 166). Three-dimensional (3D) image-capture devices that provide shape morphology along with standardized photographs (color information) were used to obtain 3D images of women. The coordinates and red‒green–blue color data on the 3D images were utilized to perform principal component (PC) analysis, and shape and color PCs were generated. A canonical variate analysis was then conducted to check for significant correlations between the shape and color PCs. As a result, 6 significant correlations were found (p < 0.05). In detail, in addition to the physical correlations (i.e., steric faces or faces with protrusion of the cheek showed greater shadows, retrognathism was related to a shadow under the lower lip and vice versa), biological correlations (fatty faces showed greater redness and remarkable marionette lines; faces with age-related sagging showed greater darkness, possibly related to cumulative ultraviolet radiation exposure of the skin; and robust mandibles and supraorbital ridges were related to thick eyebrows) were found. This insight can aid both medical and cosmetic practitioners in comprehending the intricate details conveyed by facial features, thereby facilitating more comprehensive diagnosis and treatment planning, including makeup.

## 1 Introduction

Facial appearance influences facial health perception [1], attractiveness [2], mate choice decisions [3], social interaction, and self-esteem [4]. Facial visual cues can be divided into two categories: facial shape and facial coloration. Many previous studies have determined the influence of each of these two factors on human perceptions of health. For example, facial symmetry [5], facial shape averageness [6], sexual dimorphism of facial shape [7], and facial adiposity are shape cues linked to perceived health [8]. Furthermore, darker skin under the eyes has been proven to be associated with significantly decreased perceived health [9]. Increased contrast between yellow and blue around the eyes [10] significantly decreased health status ratings. Pale skin tone, redness of the eyes and darkness around the eyes have been reported as color changes perceived as indicative of sleep deprivation [1,11,12]. A smooth and even appearance is perceived as healthy and attractive [13]. Red cheeks and high periorbital brightness are perceived as healthy [9,10]. When judging health, skin brightness and redness and yellowness on the face seem to be preferred [[14], [15], [16]], which may reflect the deposition of dietary carotenoids on the skin [[17], [18], [19], [20]]. Carotenoids may play a role in the immune function [21] and reproductive health [22], suggesting a possible link to attractiveness, as they enhance both.

In addition to perceived health, numerous studies have examined the relationship between facial features, including shape and color, and external health factors. For example, facial shape analysis also identified valid cues to aspects of physiological health such as obesity in Caucasian, Asian, and African Populations [[23], [24], [25]]. Sleep deprivation accelerates skin aging [26] and causes dark circles and scleral redness [12]. Smoking deepens the wrinkles [27], and quitting smoking lightens the skin [28]. In addition, the oxygenated state of blood is associated with health status and affects skin color [29]. Increased deoxygenation of the blood is associated with hypoxia and can cause cyanosis (blue skin), a sign of coronary artery and respiratory diseases [30]. In women, elevated levels of sex steroid hormones increase angiogenesis in the skin and cause hyperpigmentation in melasma [31]. In summary, numerous studies have shown that the actual health status of the body can impact both the facial shape and coloration, and humans can perceive these indicators as attractiveness and reflective of perceived health status.

While many studies have focused on the separate influences of facial shape and color, there is a possibility of a causal relationship between facial morphology and facial coloration, or simple correlations between the two factors that are derived from common factors. However, to date, no studies have examined the direct association between facial shape and facial color over a wide area based on our search.

The relationship between facial shape and color provides useful knowledge for clinicians in the following situations: First, knowledge regarding the relationship between facial shape and color may allow clinicians to make accurate diagnoses based on visual cues that a specific body status commonly holds in facial shape and color. Second, clinical practitioners work in interdisciplinary teams to provide comprehensive patient care. Understanding the relationship between facial shape and color allows for effective collaboration between professionals from various fields, such as dermatologists, orthodontists, plastic surgeons, orthognathic surgeons, and makeup artists. In particular, facial cosmetics can easily change facial color and have been proven to interact with attractiveness [32] and identity [33]. Optimal and comprehensive treatment plans, including fundamental morphological changes and alternative camouflage by color changes, can be proposed for each individual to tailor the treatment plans. Preventive measures for color changes such as blemishes can also be considered for specific facial shapes.

Thus far, when measuring facial colors, contact spectrophotometers have been commonly used to examine skin tones due to their accuracy [[34], [35], [36]]. The measurement of skin color using a spectrophotometer is highly reproducible and unaffected by lighting [37]. However, these small probes are not suitable for covering a wide area. Standardized high-definition photographs have also been used to evaluate skin color distribution as they cover a wide area [[38], [39], [40], [41]]. With recent technological advances, three-dimensional (3D) soft-tissue facial shape examinations with standardized high-definition photographs may provide both facial shape and color over a wide area [23].

Thus, this study examined whether the facial colors captured by standardized high-definition photographs with a wide area correlated with 3D facial morphology in Chinese women in a boundary group of young to middle-aged subjects and discussed potential biological, physical, and mathematical explanations concerning the relationship between facial shape and facial colors. We focused on a boundary group of young to middle-aged participants because this age group generally undergoes dramatic age-related changes in the face. In addition, we examined the relationship between color information obtained from a 3D image-capture device and skin colors measured by a contact-type spectrophotometer to conduct a detailed discussion. Considering that race is an influencing factor in the color and shape of the face, we focused on Han Chinese, as it is the world's largest ethnic group [42], accounting for approximately 17.5 % of the total world population.

## 2 Methods

### 2.1 Subjects

A total of 166 Han Chinese women (35–44 years old) were recruited using convenience sampling and were included in the present study. We recruited participants from among consumers of facial skin care products supplied by a company (Shiseido Co., Ltd., Yokohama, Japan) with the following inclusion criteria: 1) age range 35–44 years old; 2) lived in Shanghai for the past year; and 3) used facial skin care products with an anti-aging function supplied by the company (Shiseido Co., Ltd., Yokohama, Japan) at least five times a week. The exclusion criteria were as follows: congenital facial deformities; no facial paralysis; noticeable scars or skin disease in the neck or dentofacial regions (or history thereof); a history of any psychiatric disorder; subjectively or objectively discernible jaw dysfunction; and an apparent medical history that could affect facial shape and colors, such as a body mass index outside the normal range (18.5–25 [43]), smoking history, jaundice, or liver disease.

For the sample size calculation, first, a power analysis was conducted to determine the total sample size required to determine whether or not a correlation coefficient differed from 0. The calculations showed that a minimum of 85–194 participants were needed when the Type I error rate alpha was set at 5 %, the Type II error rate beta was set at 20 %, and the expected correlation coefficient was 0.2–0.3. Furthermore, our previous study [44] that examined facial shape differences between younger and elderly patients (average age differences of 30 years) showed that the distance between the subnasale and stomion (Sn-Sto) had an average difference of 3.5 mm with a standard deviation of 2.4 mm. Given that the age differences in the present study were set as almost 1:3, the differences for the Sn-Sto in the present study were estimated to be 1.05 mm with a standard deviation of 2.4 mm if there were linear age effects for Sn-Sto. The minimum number of participants was calculated to be 166 using the power analysis for the present study.

Data were collected from July 16th to 22nd of 2019. This study was approved by the Research Ethics Committee of Shiseido Co. Ltd. (No. C01877). Written informed consent was distributed to and signed by the participants, their parents, and/or legal guardians for study participation. Informed consent was obtained from the Research Ethics Committee of Shiseido Co., Ltd. All experiments were conducted with strict adherence to pertinent guidelines and regulations [45].

### 2.2 Data recording

• 3D shapes and colors of the face

To capture the morphological features and colors of the participants' faces, a non-invasive 3D image-capture device (VECTRA-H2 3D Imaging System; Canfield Scientific Inc., Parsippany, NJ, USA) was employed to record high-resolution 3D facial images for each participant. The capture system specifications included a 2.0-ms capture time utilizing stereophotogrammetry technology, with a 3D resolution of 1.2 mm (triangle edge length). To obtain consistent lighting conditions, the room was set as follows: no natural sunlight (no windows), consistent fluorescent light conditions, and white. Furthermore, the room temperature was maintained at 25 °C and a noiseless room was used. Each participant maintained a natural head posture without back support throughout the recording. The participants were instructed to bite their teeth lightly and keep their lips at rest. Following the manual of the 3D image-capture device, the optimum distances from the cheekbone at both sides and the subnasale were measured using a red laser light (approximately 50 cm between the lens and the participant), and three pictures from the left and right at 45° as well as from the front were taken with only flashes from the 3D image-capture device. While taking the pictures, the participants were instructed not to move. The three pictures were then stitched into a single morphology data sample and a color data sample. Colors are expressed as red-green-blue (RGB) data for each facial XYZ coordinate.

• Color of the facial skins

The primary objective of this study was to explore the correlation between color and shape across the entire face in images that were simultaneously captured by a 3D image capture device. However, when utilizing a 3D image-capture device to obtain the color data of the entire face, the potential influence of lighting conditions (even though the lighting conditions were consistent) and shadows on the measurements should be considered. To complement this, the study also utilized spectrophotometry, a reliable method unaffected by ambient lighting [37], to establish a relationship between the color data obtained from the 3D image-capture device and the actual skin colors measured using a contact-type spectrophotometer in advance. A contact-type spectrophotometer (Skin-Colorimeter CL400; Integral, Tokyo, Japan) [[34], [35], [36]] measured two points on the face (forehead and cheekbone) and two points on the body (neck and medial side of the arm). The neck measurement was utilized as a reference, considering the minimal impact of skincare products, while the measurement on the medial side of the arm served as another reference, considering the reduced exposure to both ultraviolet (UV) rays and the influence of skincare products. The color space was defined by the International Commission on Illumination (CIE) as L* for perceptual lightness (black [0] and white [100]) (L* scale) and a* and b* for the four unique colors of human vision: redness (red [+] and green [−]) (a* scale) and yellowness (yellow [+] and blue [−]) (b* scale). The CIE color space uses a set of color-matching functions called the “standard (colorimetric) observer” that describes physically produced light spectra with specific tristimulus values to consider the varying sensitivity of the eye's light receptors. This is based on experiments examining quantitative links between physically pure colors in the visible electromagnetic spectrum and physiologically perceived colors in human color vision [46,47]. For example, the human eye does not detect all colors equally, and color-sensing cones in the retina are the most sensitive to green light. The sensitivity of these special cells rapidly tails towards the blue and red parts of the spectrum. The CIE color space allows the chromaticity (an indication of the quality of a color, independent of luminescence) of a color to be plotted using two derived parameters: a* and b* [48].

To minimize potential variations and biases, we used the standardized method for measurement. In brief, before using the spectrophotometer, we calibrated the device with a standard calibration tile provided by the manufacturer. To prepare the subject for accurate and reproducible measurements, we asked the subject to equilibrate by resting for an appropriate amount of time or a minimum of 15 min before measurement. To acquire measurements, the device was held perpendicular to the skin surface, with the tip pressed against the skin under moderate pressure. The illumination conditions were set as D65.

### 2.3 Analyses

Statistical analyses were conducted using two software programs, MATLAB (MathWorks, Ltd., Natick, MA, USA) and R (http://www.r-project.org/). A comparison of 3D facial shapes and colors was performed, as outlined below.

### 2.4 Geometric morphometric analyses of 3D faces

To analyze the geometric features of the 3D faces, we utilized the coordinate system determined in a previous study (Supplementary Fig. S1) [49]. This involved defining The sagittal plane was defined using endocanthions and exocanthions, and the axial plane using the porion, subnasale, and exocanthions. The nasion serves as the reference point or origin.

For each facial surface, we employed high-resolution template meshes [49,50] and utilized a commercial software program (HBM-Rugle, Medic Engineering Co., Kyoto, Japan) to fit the meshes based on the assigned landmarks in each 3D image. The reliability of the mesh fitting has been reported as a mean absolute difference of 0.04 mm and 0.13 mm for the two examiners, respectively, indicating high reliability [51]. This fitting process generated a homogeneous model consisting of 584,063 Cartesian semi-landmarks on the wire mesh for each model. This method allowed us to extract relevant surface anatomy while removing non-relevant data, resulting in 3D surface data that provided sufficient detail for quantitative assessment while maintaining manageable file sizes (Supplementary Fig. S2). The 584,063 Cartesian semi-landmark coordinates were then subjected to geometric morphometric analysis.

### 2.5 Dimensional reduction of the shape and color information

To reduce the dimensional information of the facial shapes and colors of the subjects, we performed principal component analysis (PCA) for the 584,063 coordinates (XYZ) and corresponding color data (RGB) of the surface model. The significant shape principal components (sPCs) and color principal components (cPCs) were determined based on the Kaiser criterion (sPCs and cPCs with eigenvalues greater than one were included).

### 2.6 Colors from the 3D image-capture device and actual skin colors from the contact-type spectrophotometer

The aforementioned cPCs and L*a*b* data from the spectrophotometer were examined using the correlation coefficients. Statistical significance was set at a p-value of 0.05.

### 2.7 Relationships between facial shapes and colors

First, all combinations of sPCs and cPCs were examined for significant correlations. A canonical variate (CV) analysis was then conducted to determine the maximum correlations between sPCs and cPCs [23]. Significance was set at a p value of <0.05, and a coefficient correlation value of r > 0.5.

## 3 Results

### 3.1 Preprocessing of facial shapes and colors from a 3D image-capture device

**Fig. 1:**
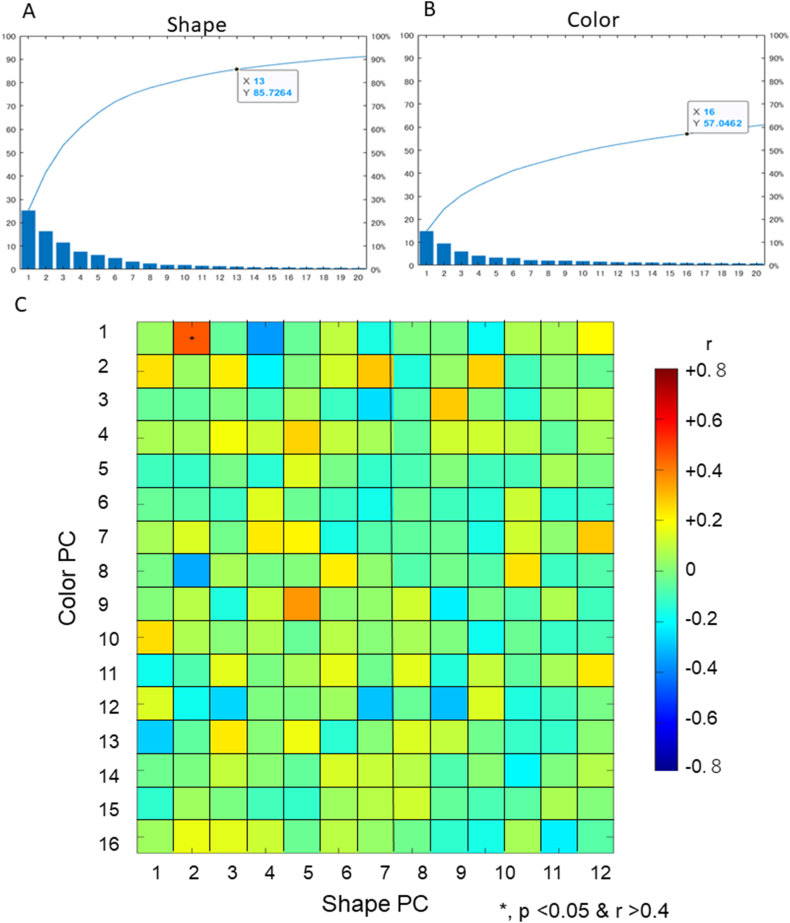
**Scree plot of the principal component (PC) analysis for shape (A) and color (B) and the correlation coefficients between them (C).** Thirteen shape PCs explain 85.7 % of the variations from total samples, while 16 color PCs explain 57.0 % of total samples.

PCA decomposed the facial shape into 13 significant facial shape PCs (sPCs 1–13, Fig. 1A, and Supplementary Fig. S3), based on the Kaiser criterion. These factors were found to explain 85.7 % of the total variation in facial shapes. For facial colors, 16 significant color PCs (cPCs 1, 2, …, 16; Fig. 1B and Supplementary Fig. S4) were extracted. cPCs 1, 2, …, and 16 represented 57.0 % of the total variation in facial color. The interpretations of sPCs and cPCs are presented in Table 1, Table 2, respectively.

**Table 1:** Interpretation of the sPCs.

Shape	Interpretation for the positive sPC value
sPC1	Smaller lower facial width and a retruding chin with the thick lower lip
sPC2	Less protrusion of the forehead with a greater width of the face
sPC3	Protrusion of the chin with a short face
sPC4	The greater height of the forehead with upward slated eyes
sPC5	Asymmetry of the face
sPC6	Robust mandible (greater width and protrusion of the mandible)
sPC7	Protruded nose and midface
sPC8	Asymmetry of the face
sPC9	Protrusion of the lower cheek with greater lower face and a downward slant of the oral commissures
sPC10	Cheek protrusion with a smaller lip width
sPC11	Flat cheek
sPC12	Protrusion of the upper lip and mastoid region of the cheek
sPC13	Protrusion of the eyes, noses, and chin

sPC, shape principal component.

**Table 2:** Interpretation of the cPCs.

Color	Interpretation for the positive cPC value
cPC1	Less shadowed facial outlines
cPC2^b^	The shadow of the mastoid regions, yellowness (+b*) of the facial skin
cPC3	Less shadow of the chin and less remarkable nasolabial fold
cPC4^a^	Whiteness (-L*), the tendency of the cold color tendency (-a*, -b*) especially at the cheekbone, indicating a relation to fewer cumulative UV exposure to the face. Blueness (-b*) of the skin was also observed in the non-UV exposed medial side of the arm.
cPC5	Asymmetry color of the face
cPC6^b^	Darkness around the mouth and redness (a*) of the facial skin of the cheekbone.
cPC7^a^	Less shadow of the lateral part of the cheek, darkness (-L*) of the facial skin related to body skin colors.
cPC8^b^	Less darkness of the mouth, especially below the lower lip; yellowness (b*) of the skin at the forehead and cheekbone.
cPC9^b^	Thin eyebrows and darker colors around the noses and lower lips; yellowness (b*) of the skin at the forehead and cheekbone
cPC10^a^	Fewer marionette lines; Whiteness (+L*), yellowness (+b*), and greenness (-a*) of facial skin related to the yellowness (+b*) of body skin colors. This indicates a relation to less blood flow or smaller hemoglobin.
cPC11	Less dark portions below the eyebrows and darker upper and lower lips
cPC12	The shadow under the lower lip
cPC13^b^	Redness (+a*) of the facial skin. This indicates a relation to greater blood flow or greater hemoglobin.
cPC14	Greater darkness around the eyes (dark circles under the eyes) and less shadow of the chin
cPC15	Less darkness around the eyes and less shadow of the chin
cPC16	Less shadow/darkness of the cheek

cPC, color principal component.

a , cPCs related to skin color of the face and body (Type 1).

b cPCs related to skin color of the face but not related to the body (Type 2); no mark, cPCs not related to skin color of the face or body (Type 3).

### 3.2 Colors from a 3D image-capture device and actual skin colors from a contact-type spectrophotometer

**Fig. 2:**
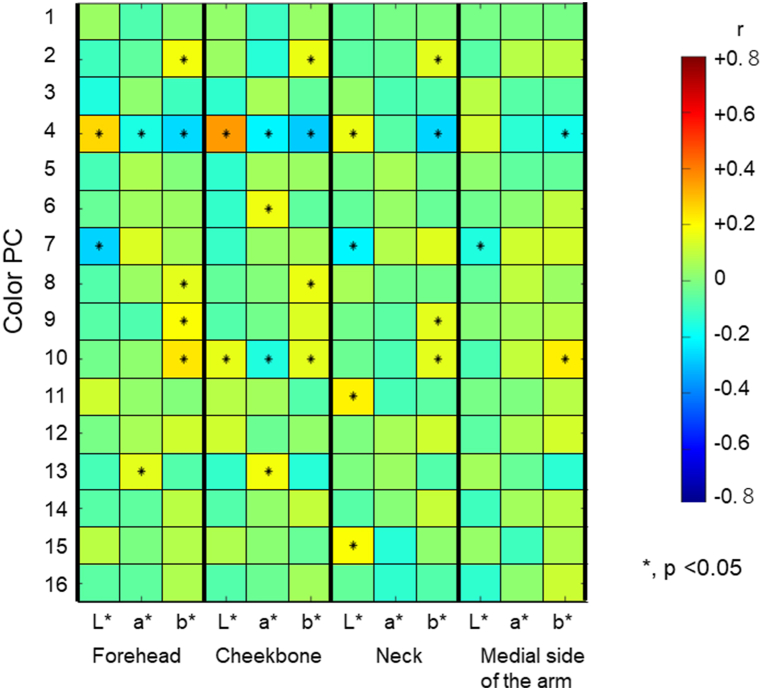
The correlation coefficients between L*a*b* values of the skin (at the forehead, cheekbone, neck, and medial side of the arm) and color principal components (cPCs). *indicates a significant correlation (p < 0.05).

A comparison of the skin colors measured by spectrophotometry and cPCs (colors obtained by the 3D-image-capture system) showed 30 significant correlations between them (Fig. 2, p < 0.05). Among these, cPCs related to the medial side of the arm (underside of the upper arm) were considered to reflect the entire body of the subjects, as the underarms are considered to be relatively less exposed areas of the skin and are not influenced by sunburn.

Based on the correlation with the above spectrophotometry in the face and body, cPCs were classified into three categories: type 1, cPCs related to skin colors of the face and body; type 2, cPCs related to skin colors of the face but not related to the body; and type 3, cPCs not related to skin colors of the face or body. Type 3 cPCs were more likely to be related to shadowing (e.g., shadowed facial outlines and nasolabial folds).

A previous study [52] demonstrated that an increase in the b* scale component and decrease in the L* scale component indicated skin darkening caused by cumulative UV exposure. Among the cPCs related to skin color (i.e., Types 1 and 2), a negative value of cPC4 satisfied this condition (decrease in the L* scale component and increase in the b* scale component), suggesting that a smaller cPC4 may represent cumulative UV exposure, and skin colors of the cheekbone showed the greatest correlation with cPC4 (r = +0.5 (p < 0.05). The positive value for cPC7 also indicated a correlation with a decrease in the L* scale component, but this value was correlated with the forehead, neck, and medial side of the arm (r = -0.2 − −0.4, p < 0.05) and not with the cheekbone, suggesting that cPC7 is unlikely to be related to UV exposure. cPCs 2, 8, 9, and 10 were weakly related to yellowness of the forehead, cheek, and neck (r = +0.2, +0.3, p < 0.05). Furthermore, a greater hemoglobin level was considered to be expressed as a greater a* and smaller L* [[53], [54], [55]], and the negative value of cPC10 and positive value of cPC13 were considered to be related to the amount of hemoglobin.

### 3.3 Correlations between the facial shapes and colors

The correlation between sPCs and cPCs was examined (Fig. 1C). While most of the correlations showed coefficient correlations <0.4, a significant correlation was found between sPC 2 and cPC 1 (p < 0.05, r > 0.4). A less shadowed facial outline (cPC1) was related to less protrusion of the forehead (sPC2).

### 3.4 Canonical correlations between the facial shapes and colors

To examine the total correlations between facial shapes and colors, CV analysis was conducted. This analysis of the facial characteristics and colors showing the maximum correlation between them detected six significant canonical correlations between facial shape and color (CV1 to CV 6, p < 0.05, r > 0.5, Table 3).

**Table 3:** Canonical variates conducted to identify any significant correlations between shape and color data of the face.

	r	Wilks	df1	df2	F	pF	chisq	pChisq	dfe
**CV1**	**0.81**	**0.01**	**208.00**	**1419.33**	**4.20**	0.00**	**730.79**	0.00**	**208.00**
**CV2**	**0.78**	**0.02**	**180.00**	**1326.10**	**3.65**	0.00**	**571.99**	0.00**	**180.00**
**CV3**	**0.71**	**0.06**	**154.00**	**1231.67**	**3.12**	0.00**	**434.53**	0.00**	**154.00**
**CV4**	**0.67**	**0.11**	**130.00**	**1136.00**	**2.73**	0.00**	**330.88**	0.00**	**130.00**
**CV5**	**0.61**	**0.21**	**108.00**	**1039.02**	**2.33**	0.00**	**241.87**	0.00**	**108.00**
**CV6**	**0.55**	**0.33**	**88.00**	**940.62**	**1.97**	0.00**	**172.21**	0.00**	**88.00**
CV7	0.48	0.48	70.00	840.64	1.63	0.00**	116.98	0.00**	70.00
CV8	0.40	0.62	54.00	738.85	1.34	0.05	76.73	0.02*	54.00
CV9	0.38	0.74	40.00	634.83	1.14	0.26	50.27	0.13	40.00
CV10	0.27	0.87	28.00	527.83	0.77	0.80	24.81	0.64	28.00
CV11	0.22	0.93	18.00	416.26	0.56	0.92	12.56	0.82	18.00
CV12	0.12	0.98	10.00	296.00	0.30	0.98	4.06	0.94	10.00
CV13	0.07	1.00	4.00	149.00	0.17	0.96	1.19	0.88	4.00

CV, canonical variates; r, sample canonical correlation; Wilks, Wilks' lambda (likelihood ratio) statistic; df1, degrees of freedom for the chi-square statistic and numerator degrees of freedom for the F statistic; dfe, denominator degrees of freedom for the F statistic F; Rao's approximate F statistic for the null hypotheses H_0_^(k)^; pF, right-tail significance level for F; chisq, Bartlett's approximate chi-squared statistic for H_0_^(k)^ with Lawley's modification; pChisq, right-tail significance level for chisq, where d null hypothesis H_0_^(k)^ that the (*k*+1)st through *d*th correlations are all zero for *k* = 1, …,*d*-1. Bold values represent CVs with p < 0.05 and r > 0.5.

**Fig. 3:**
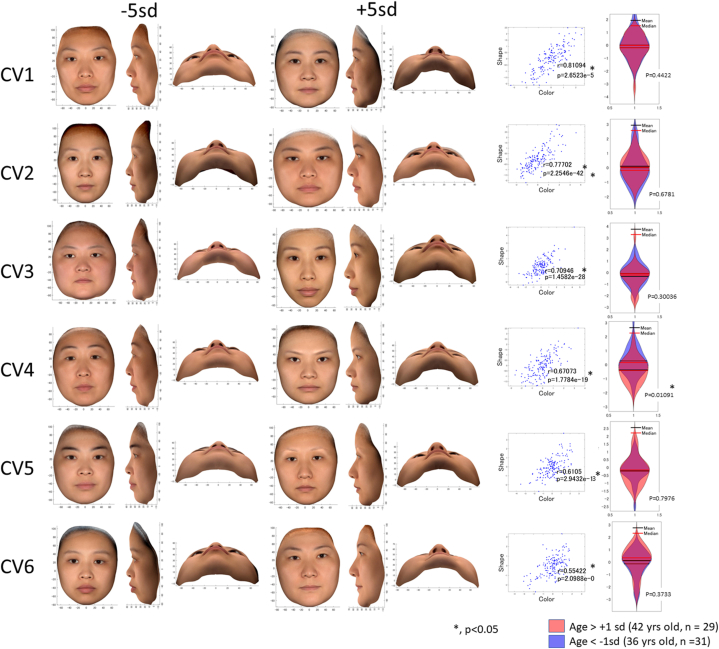
**Results of a canonical variate (CV) analysis.** A significant correlation was found in the first to sixth CVs (p < 0.01). The results were expressed as combined color and shape data. The left first to third columns indicate −5 standard deviation (sd) for the representative shape and color data variation; left fourth to sixth columns, +5 sd; right second columns indicate the subject plot for color synthetic variables (x-axis) and the shape color synthetic variables (y-axis) for each CV. *r* denotes the canonical correlation for each CV. The right first column shows the comparison of the color synthetic variables between a group of patients of >42 years of age (>+1 sd) and a group of patients of <36 years of age (<–1 sd). This figure was created mathematically and does not contain any personally identifiable information.

**Fig. 4:**
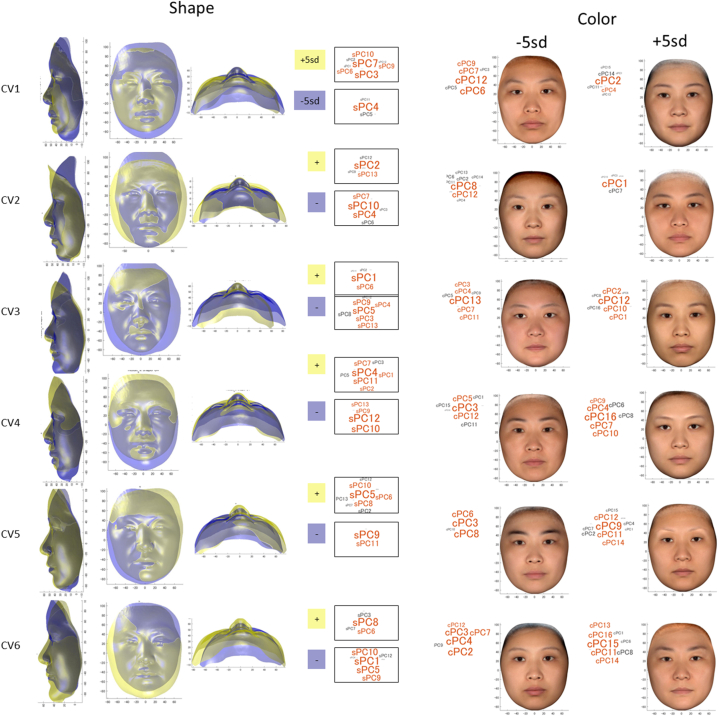
**Results of a canonical variate (CV) analysis.** A significant correlation was found in the first to sixth CVs (p < 0.01). The results are expressed as separate figures for color and shape data. Canonical loadings for color principal components (cPCs) and shape PCs (sPCs) are represented as word clouds, where the relatively large and orange words were the most highly weighted PCs when creating synthetic variables in the canonical variate (CV) analysis, while the smaller and black words were the secondary weighted PCs. This figure was created mathematically and does not contain any personally identifiable information.

Both CVs 1 and 2 were related to the shadow of the face, and the flat cheek was related to less shadow of the facial outlines (see the bottom view of Fig. 3, Fig. 4). Specifically, with a positive direction of CV1, protrusion of the chin, nose, and midface (+sPC3, +sPC7) with a short vertical facial height, including the forehead height, with downward slanted eyes (+sPC3, -sPC4) (i.e., a brachycephalic face) were significantly associated with a greater shadow of the face line (+cPC2). In contrast, in the negative direction of CV1, retrusion of the chin with a long facial height (i.e., skeletal 2 dolichocephalic face) was significantly related to a greater shadow under the lower lip (+cPC12), darkness around the mouth, and redness of the facial skin of the cheekbone (+cPC6).

With a positive direction of CV2, greater facial width (+sPC2), smaller forehead height with downward slanted eyes (-sPC4), and flat cheek with greater lip width (-sPC10) were significantly associated with less shadowing under the cheek (+cPC1). With a negative direction of CV2, a smaller facial width, greater height of the forehead, and protruding cheek showed a greater shadow under the cheek.

In the positive direction of CV3, a vertically, horizontally, anteroposteriorly small lower face with a thick lower lip with an upward slant of the oral commissures (+sPC1, -sPC9) with facial asymmetry (-sPC5) was associated with a greater shadow under the lower lip (+cPC12). In contrast, a greater width of the lower face, which may represent a fatty face or mandibular prognathism, with a thin lower lip and downward slant of the oral commissures, was related to redness of the facial skin (+cPC13, -cPC10), possibly due to a greater hemoglobin amount or greater blood flow, as well as marionette lines (-cPC10). In the positive direction of CV4, protrusion of the nose with a flat cheek and upper lip, greater lip width (-sPC10, -sPC12), smaller lower facial height (+sPC1), and greater forehead height with upward slated eyes (+sPC4) were associated with a smaller shadow under the lower lip (-cPC12) and less darkness of the cheek (+cPC16). In contrast, retrusion of the nose with a protruding cheek and upper lip, greater lower facial height, and smaller forehead height were related to darkness of the cheek skin, which was considered to be due to cumulative UV exposure (-cPC4) and a greater nasolabial fold with marionette lines (-cPC3, -cPC10). The CV4 value was significantly greater in the younger subgroup (<36 years old) than in the older subgroup (>42 years old), indicating that negative values were related to facial aging.

In the positive direction of CV5, asymmetry of the face (+sPC5) and retrusion of the lower cheek with a vertically and horizontally smaller lower face (+sPC6, -sPC9) were related to thin eyebrows and darker colors around the nose and lower lip (-cPC8, +cPC9). In contrast, in the negative direction of CV5, protrusion of the lower cheek with a vertically and horizontally greater lower face (-sPC6, +sPC9) (i.e., robust mandibles and/or greater masseters) as well as a greater supraorbital ridge (+sPC9) and greater lip width (-sPC10) were associated with thick eyebrows and a less remarkable nasolabial fold (+cPC3).

In the positive direction of CV6, protrusion of the chin (+sPC6, -sPC1), which represents a concave profile, with thin lips (-sPC1) and asymmetry of the face (+sPC8) were related to fewer dark circles around the eyes and less shadow of the chin (+cPC15). In contrast, a convex profile with thick, protruding lips was related to darkness around the eyes and a greater shadow under the lower lips (+cPC2, +cPC3, +cPC4, +cPC7, and-cPC11).

## 4 Discussion

This study examined the relationship between facial morphological characteristics and facial color. The relationships between the shape and colors were mainly as follows: a steric face or a face with protrusion of the cheeks showed a greater shadow (CVs 1 and 2); a fatty face with a greater facial width, thin lips, and chin protrusion with downward slanted lip commissures showed greater redness and remarkable marionette lines (CV3); age-related sagging faces showed greater darkness, possibly due to cumulative UV exposure (CV4); robust mandibles and supraorbital ridges were related to thick eyebrows (CV5); and protrusion of the chin, representing a concave profile, with thin lips related to less dark circles around the eyes and less shadowing of the chin (CV6).

Regarding CVs 1 and 2, steric faces, which are easily recognized in the bottom view, tended to show greater shadowing (physical correlations). This can be explained mathematically as the shadow being formed by the angles between the direction of light and the normal vector of the facial surface.

Regarding CV3, fatty facial shape was related to redness of the facial skin (greater blood flow and greater hemoglobin), indicating biological correlations. Previous studies [[56], [57], [58]] have demonstrated a consistent association between individuals exhibiting a higher body fat proportion and the manifestation of facial characteristics typically associated with adiposity. Another study [59] showed that individuals with increased cheek fat had increased visceral fat. Individuals displaying fuller cheeks may face an increased susceptibility to metabolic complications stemming from obesity compared to individuals without this particular facial trait. Accordingly, reddening of the face is considered to be caused by hypertension, which is related to metabolic disorders. Furthermore, pathophysiological dysfunction, in which the body loses metabolic control, can lead to skin diseases, which can change facial texture [[59], [60], [61], [62], [63]].

From an orthodontic clinical perspective, a negative CV3 value indicated mandibular prognathism (Class III malocclusion) with a low mandibular plane angle. The negative CV3 value also aligns with other soft tissue characteristics related to Class III malocclusion, such as downward-slated lip commissures and thin lips, in line with these prior findings [64,65]. Interestingly, it has been reported that obesity (fatty face) tends to involve Class III malocclusion traits (i.e. greater mandibular length and lower mandibular plane angle compared to a control group) [24,25,66,67]. Thus, it can be said that a negative CV3 value is related to all of these Class III and obesity characteristics.

In CV3, remarkable melomental folds (marionette lines), which are long vertical lines surrounding the chin on both sides, were determined as color data. This line has been reported to be created by the mandibular ligament and comprises fatty tissue of the cheeks or sagging of the cheeks related to aging and has been considered an esthetically important issue for patients [68,69]. To our knowledge, the present study is the first to describe the relationships between marionette lines and Class III obesity. Marionette lines have traditionally been considered a non-orthodontic concern, and there is little research on the potential improvement of marionette lines through orthodontic treatment. Orthodontic treatment for Class III malocclusion typically involves either camouflage (inclination of mandibular incisors) or surgical intervention (setback of mandibles). Camouflage treatment involves inclining the upper incisors labially and lower incisors lingually; thus, the influence on the facial soft tissues is limited, while orthognathic surgery can change soft tissue shapes. Should surgical intervention prove efficacious in rectifying marionette lines, individuals desiring the eradication of such lines may choose surgery as their preferred option over camouflage treatments. Alternatively, camouflage treatment using makeup to conceal them can be recommended for those who wish to temporarily mask these lines. Esthetic surgery, such as face lifting [70] or weight reduction, can also be advised. The present study warrants further research on marionette lines obtained through orthodontic treatment.

CV4 seems to represent the shape and color pattern related to aging, and the shape values of CV4 are significantly related to age groups (biological correlations). Furthermore, skin color was significantly related to color values at CV4, especially at the cheekbone. Previous studies have shown that the lightness of the face decreases as age increases, which is related to a decrease in redness, an increase in yellowing, and a decrease in lightness, while skin coloration is mainly determined by hemoglobin (red), carotene (yellow), and melanin (brown) [71]. These changes are considered to be due to decreased blood flow, thickening of the stratum corneum, hyperpigmentation, carbonylation of skin proteins, glycation, thickening of the stratum corneum, and oxidation, which increase with age [72]. The absorption of light due to the thickness and surface condition of the stratum corneum (texture, wrinkles, etc.) can also be related to skin coloration.

Our CV4 findings concerning colors correspond well with previous skin color results [71]. The decrease in lightness was particularly noticeable in the area along the cheekbones, from the temples to the center of the cheeks. Regarding the aging shape characteristics, the shape values of CV4 also corresponded to those reported in a previous study [44]. Age-related changes in facial morphology can be categorized into three main patterns. First, there are vertical downward changes, which involve the appearance of larger bags under the eyes, a reduction in the height of the eye fissures, an increase in the lower facial height, lengthening of the subnasal region, and downward displacement of the eyes, cheeks, and mouth. Second, there are transverse widening changes, characterized by an increased width of the forehead and lower face as well as a greater nasal width. Finally, protrusive changes are observed in the infraorbital region, accompanied by a straight or convex profile in the subnasal region [44]. Thus, CV4 was considered to involve the correlation of colors with shapes that have common factors related to aging rather than the influence of shapes on colors.

Regarding CV5, where a robust mandible and supraorbital ridge were shown to be related to thick eyebrows, interpretation is slightly difficult, but sex hormones may be involved (biological correlations). Sex hormones have a significant impact on facial shape [73]. Previous research suggests that higher testosterone levels in women are linked to facial features that are commonly associated with masculinity, such as an increased height-to-width ratio of the midface [73]. Furthermore, a twin study found that female infants with a male twin, who likely experienced higher levels of fetal testosterone, had more prominent masculine facial features than those with a female twin [74]. Previous studies showed that maleness of the face [49,75] was reflected by a greater lower face height, a protruded forehead, a flat cheek in the infraorbital region but more prominence of the cheek in the parotid-masseteric region, and an anteroposteriorly greater nose when compared with female counterparts. Thus, the negative shape of CV5 appeared to correspond well with the maleness of the face. In women, increased androgen bioavailability is commonly observed in polycystic ovary syndrome (PCOS) [76], a highly prevalent endocrine disorder among young women, with an estimated occurrence of 12 % in premenopausal women. This syndrome is characterized by several clinical features including hyperandrogenemia, excessive hair growth (paradoxical hypertrichosis), reproductive dysfunction, insulin resistance, and hyperinsulinism [77]. Based on these findings, CV5 can be explained by sex-hormone differences. Similar to other factors that may be related to eyebrows, hypo- and hyperthyroidism typically shows hair loss of the eyebrows in cases with a positive CV5 value. However, eyebrow shape and density can be easily changed by the subject's maintenance, making it difficult to specify the factors that primarily affect these results, which is a limitation of the present study.

Common characteristics of the convex-type profile with protruding thick lips were related to the shadow under the lip in CV1, CV3, and CV6. Previous studies examining two-dimensional (2D) facial profiles [64,71] have shown that convex-type profiles tend to have a greater height of the lip vermilions, which is well explained in -CV1, +CV3, and -CV6, and is related to bimaxillary-protrusion or incompetent-lips appearance. The shadow of the lower lip can be defined mathematically, but this is the first study to describe the exact facial appearance with a focus on color. Traditionally, the diagnosis of a convex-type profile or bimaxillary protrusion has been made through lateral views, and incompetent lips refer to lips that cannot be closed without exertion. The present study revealed a noteworthy observation of excessive shadowing beneath the lower lips in individuals with bimaxillary protrusion or incompetent lips. This phenomenon, which is visible in frontal views, contributes to an abnormal appearance and is indicative of bimaxillary protrusion or incompetent lips. This study emphasizes the importance of considering color information beneath the lower lip during diagnostic assessments by orthodontists.

The treatment for bimaxillary protrusion or incompetent-lips appearance includes orthodontic treatment with premolar extraction [50], molar intrusion [78], anterior segmental osteotomy [79], set-forward surgery of the mandibles [80], chin augmentation [81], and genio-pasty. However, it is crucial to note that further research is needed to explore the changes in color beneath the lower lips. Notably, chin augmentation may not effectively address shadows under the lower lip, as this shadow does not undergo significant alterations solely through chin augmentation. These findings underscore the potential utility of color change information in guiding treatment selection for patients undergoing orthodontic treatment. Additional research is warranted to enhance our understanding of the nuances associated with color changes beneath the lower lips and refine treatment approaches accordingly.

In contrast, individuals exhibiting traits of +CV1, -CV3, and +CV6 present with thin lips and a lack of shadow beneath the lower lips. This absence of shadows under the lower lip is considered to be related to a shallow mentolabial sulcus and concave-type profile, which are commonly associated with mandibular prognathism or Class III malocclusion. A previous study examining attractiveness in each malocclusion pattern [82] showed that a thin mentolabial sulcus was a significant factor affecting conceptions of facial beauty in patients with Class III malocclusion and explained 45 % of the variation in facial attractiveness. Research indicates that cosmetic makeup can enhance perceived attractiveness in both women [32,[83], [84], [85]] and men [86]. Furthermore, facial contrast, which is often achieved through makeup techniques [87], plays a crucial role in enhancing attractiveness. As stated above, Class III malocclusion can be treated with surgery or orthodontic camouflage. If patients undergoing orthodontic camouflage treatment for Class III malocclusion have thin lips and showed no shadow under the lips (as shown + CV1, -CV3, and +CV6), creating a dark shadow area under the lower lips by makeup techniques (known as “shading”) can camouflage the Class III appearance from the frontal view, proving useful for such patients. Patients with a cleft lip and palate tend to have a concave profile, and makeup for these patients to create a more attractive face can be proposed after orthodontic treatment if patients have these characteristics. The face is an important element for acquiring and maintaining a patient's self-esteem, and achieving social acceptance and appropriate control of facial appearance is an important issue in both clinical and general situations. Since practitioners in the orthodontic, surgical, cosmetic, and dermatological fields can change the appearance of the face, they need to fully understand the relationship between facial morphology and color to determine how best to manage these parameters. In particular, cosmetics are an important option when surgical correction of the facial form is difficult or inappropriate, and a combination of different approaches can also be selected to achieve the desired outcome. For example, as revealed in the present study, excessive lighting of the chin area, presence of marionette lines, and absence of a shadow under the lower lip are associated with the facial morphology of mandibular prognathism. While surgical orthodontic treatment may be useful for fundamentally correcting this condition, cosmetics may also be used to camouflage morphological abnormalities by changing chin color. Nutritional guidance may also be useful for managing redness due to obesity. In this respect, the present study provides useful and fundamental information on providing appropriate guidance to clients/patients requesting to change their facial appearance.

This study has several limitations. First, the sample size of our analysis was limited. Second, the age range was limited to 35–49 years old, and the ethnicity was predominantly Chinese. Our study did not incorporate socioeconomic factors or consider the potential impact of facial care products on skin color, which can vary significantly across different socioeconomic strata. Readers should consider that these sample characteristics might limit the applicability of our findings to other populations. Third, we utilized convenience sampling from cosmetic customers, a method that inherently limits access to a broader population. In particular, skin products caused errors in the results of this research. This underscores the need for future research that employs random sampling methods to ensure representative data collection. Fourth, this study primarily included normal-weight participants, which may have influenced the results and their generalizability to individuals with different weight statuses. Therefore, future research with larger and more diverse samples encompassing a wider age range, ethnicity, and weight categories is needed to validate our findings. Including a more diverse sample would strengthen the applicability of our findings in future research. The physical correlations observed, such as greater shadows on steric faces or faces with protrusion of the cheek, and the relationship between retrognathism and shadows under the lower lip, can be regarded as fundamental phenomena, applicable across different ethnicities and age groups. The biological correlations (fatty faces showed greater redness and remarkable marionette lines; faces with age-related sagging showed greater darkness, possibly related to cumulative UV radiation exposure of the skin; robust mandibles and supraorbital ridges were related to thick eyebrows) should be confirmed in larger populations. Finally, when discussing potential explanations for the observed correlations, it is crucial to emphasize the study's cross-sectional design, which precludes drawing causal inferences. It is important for readers to understand that this study establishes correlations, not causation, between facial shape and color, necessitating careful attention to prevent misinterpretation.

## 5 Conclusion

The present study revealed that facial color correlated with 3D facial morphology in Chinese women in their 30s and 40s. The relationships between the shape and color were mainly as follows: a steric face or a face with protrusion of the cheek showed a greater shadow; a fatty face showed greater redness and remarkable marionette lines; an age-related sagging face showed greater darkness, possibly due to cumulative UV exposure of the skin; robust mandibles and supraorbital ridges were related to thick eyebrows; and protrusion of the chin, representing a concave profile, with thin lips was related to less dark circles around the eyes and less shadowing of the chin.

## Data availability statement

The data that support the findings of this study are available from the corresponding author upon reasonable request.

## CRediT authorship contribution statement

**Chihiro Tanikawa:** Writing – review & editing, Writing – original draft, Visualization, Validation, Supervision, Software, Methodology, Investigation, Funding acquisition, Formal analysis, Conceptualization. **Haruna Yamanami:** Writing – review & editing, Validation, Supervision, Resources, Project administration, Methodology, Investigation, Funding acquisition, Formal analysis, Data curation, Conceptualization. **Megumi Nagashima:** Writing – review & editing, Validation, Supervision, Resources, Methodology, Investigation, Funding acquisition, Formal analysis, Data curation. **Seiko Matsumoto:** Writing – review & editing, Formal analysis, Data curation.

## Declaration of competing interest

The authors declare the following interest: “This work was partially supported by Shiseido Co., Ltd. HY, MN, and SM are employed by Shiseido Co., Ltd. Further, the funder (Shiseido Co., Ltd.) and 10.13039/501100004206Osaka University have submitted a patent application based on the results of the present study. This does not alter our adherence to the journal policies on sharing data and materials. “
