# Supplementary material for: Association between the three-dimensional facial shape and its color in a boundary group of young to middle-aged Asian women
Source: Heliyon. 2024 May 28;10(11):e32033. doi: 10.1016/j.heliyon.2024.e32033 (PMC11176851; doi:10.1016/j.heliyon.2024.e32033)
Supplement: Multimedia component 1 [file mmc1.docx]

***Supplementary Fig S1.*** ***Coordinate system and landmarks employed in the present study.*** Please see a reference ^1^ for details.


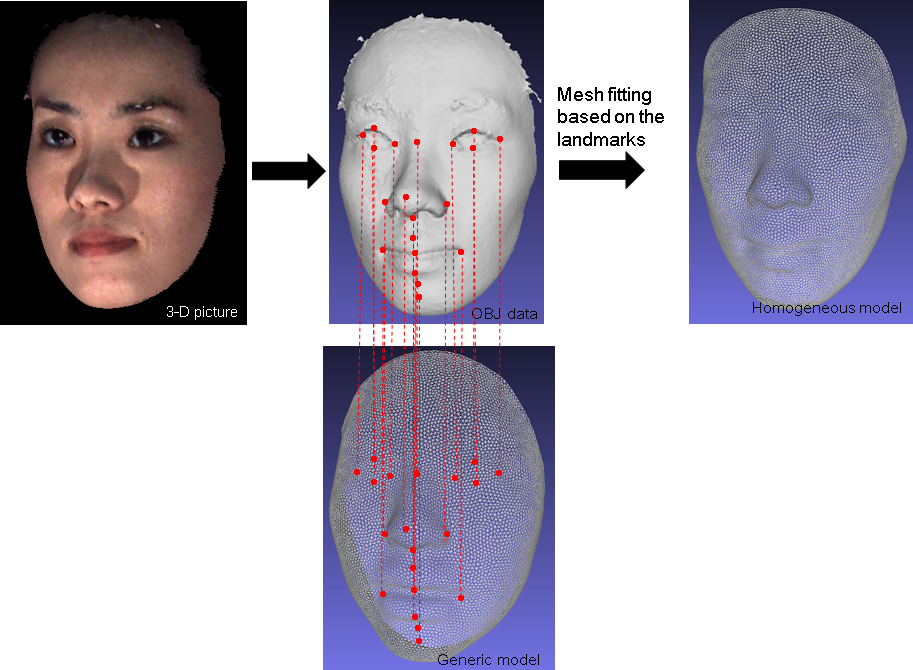


***Supplementary Fig S2. Schematic illustration of the wire mesh fitting and the point cloud of the face that were examined ^2^.***

For each facial surface (top left), fitting of high-resolution template meshes or a generic model (bottom center) was performed using commercial software (HBM-Rugle, Medic Engineering Co., Kyoto) based on the landmarks assigned to each 3D image (top center). This method automatically generated a homogeneous model (top right) that consisted of 584,063 points (i.e., fitted mesh or semi-landmark nodes) on the wire mesh for each model with landmark anchors. The semi-landmark nodes on the wire mesh of the homogeneous model generated for each face were used for further calculation.


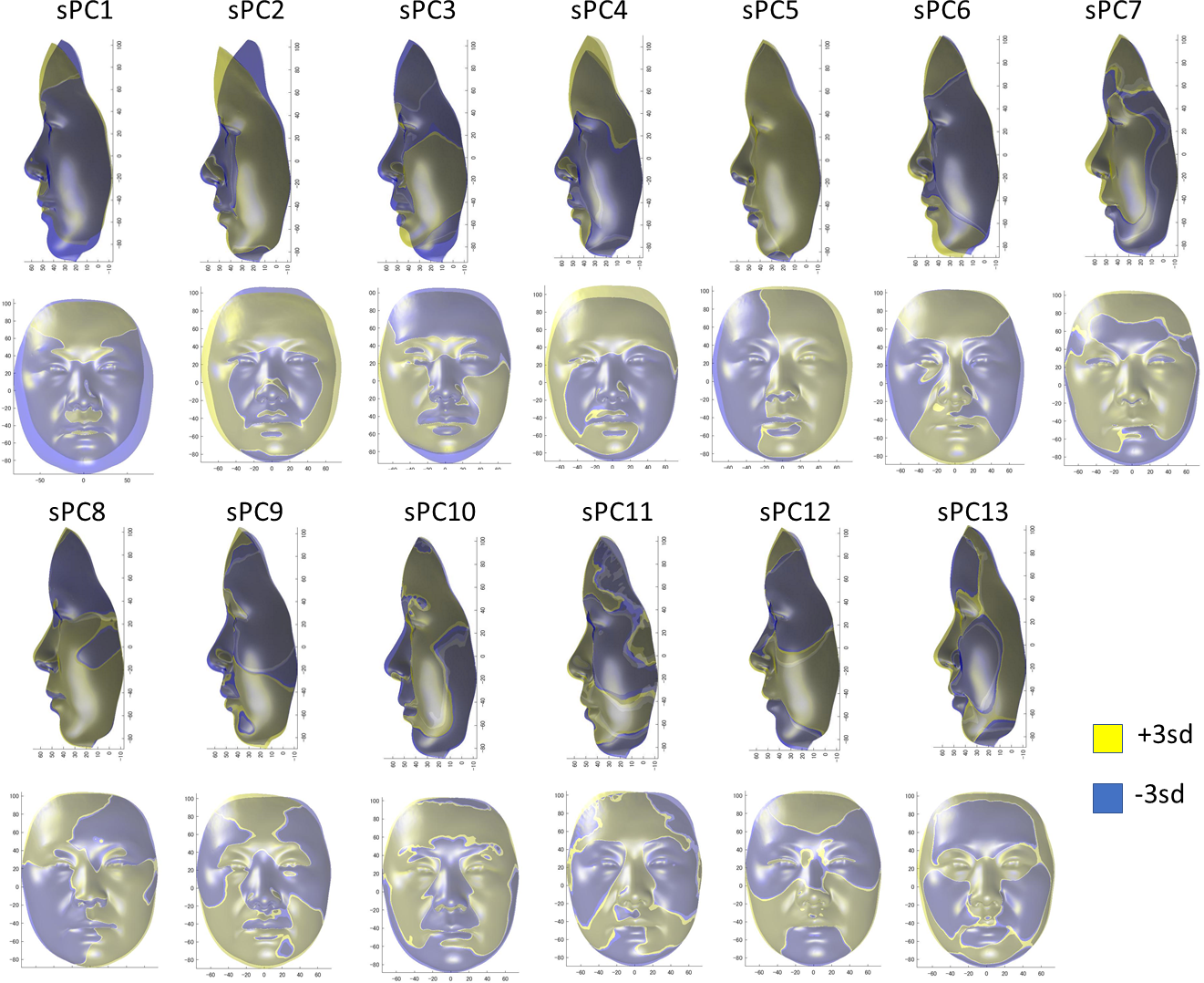


**Supplementary Fig S3.** Shape principal components (sPCs) 1 to 13. Yellow indicates the +3 standard deviation (σ); blue, -3 σ. sPCs 1 to 13 accounted for 85.7% of the total variation of the faces in the samples.

**
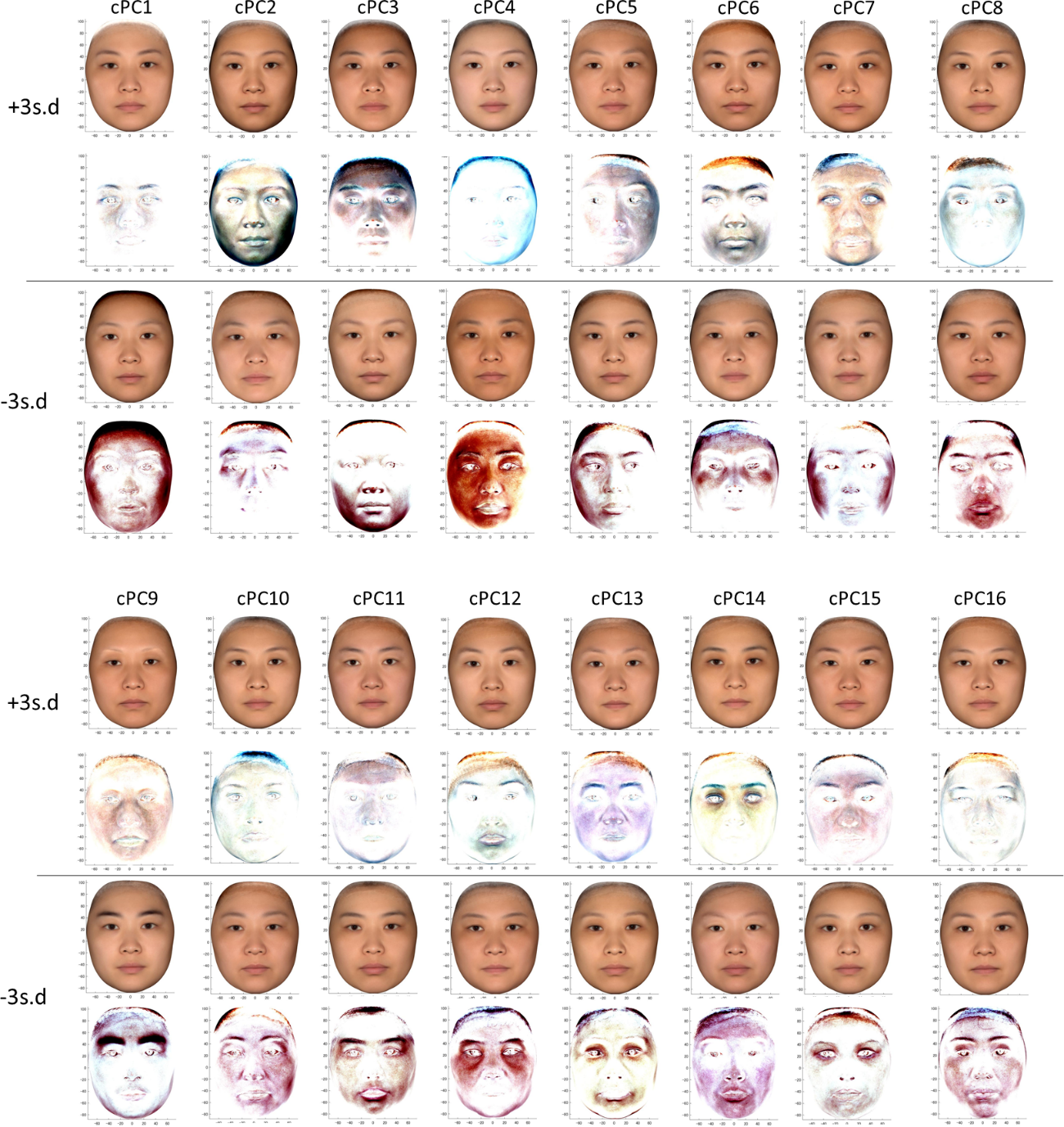
**

**Supplementary Fig S4.** Color principal components (cPCs) 1 to 16. The upper top column indicates the +3 standard deviation (σ); the upper second top column, -3 σ. cPCs 1 to 16 accounted for 57.0% of the total variation of the faces in the samples.

**References**

1 Tanikawa, C., Zere, E. & Takada, K. Sexual dimorphism in the facial morphology of adult humans: A three-dimensional analysis. *Homo* **67**, 23-49, doi:10.1016/j.jchb.2015.10.001 (2016).

2 Tanikawa, C., Akcam, M. O. & Takada, K. Quantifying faces three-dimensionally in orthodontic practice. *J Cranio Maxill Surg* **47**, 867-875, doi:10.1016/j.jcms.2019.02.012 (2019).
